# Ecological and network analyses identify four microbial species with potential significance for the diagnosis/treatment of ulcerative colitis (UC)

**DOI:** 10.1186/s12866-021-02201-6

**Published:** 2021-05-04

**Authors:** Wendy Li, Yang Sun, Lin Dai, Hongju Chen, Bin Yi, Junkun Niu, Lan Wang, Fengrui Zhang, Juan Luo, Kunhua Wang, Rui Guo, Lianwei Li, Quan Zou, Zhanshan (Sam) Ma, Yinglei Miao

**Affiliations:** 1 Computational Biology and Medical Ecology Lab, State Key Laboratory of Genetic Resources and Evolution, Kunming Institute of Zoology, Chinese Academy of Sciences, Kunming, China; 2 Kunming College of Life Sciences, University of Chinese Academy of Sciences, Kunming, China; 3 Department of Gastroenterology, The First Affiliated Hospital of Kunming Medical University, Yunnan Institute of Digestive Disease, Kunming, Yunnan China; 4 Faculty of Science, Kunming University of Science and Technology, Kunming, China; 5 College of Mathematics, Honghe University, Mengzi, Yunnan Province China; 6 Department of General Surgery, The First Affiliated Hospital of Kunming Medical University, Yunnan Institute of Digestive Disease, Kunming, Yunnan China; 7 Institute of Fundamental and Frontier Sciences, University of Electronic Science and Technology of China, Chengdu, China; 8 Center for Excellence in Animal Evolution and Genetics, Chinese Academy of Sciences, Kunming, China

**Keywords:** Inflammatory bowel disease, Ulcerative colitis, Mucosal microbiome, Species diversity, Species co-occurrence network, Core/periphery network

## Abstract

**Background:**

Ulcerative colitis (UC) is one of the primary types of inflammatory bowel disease (IBD), the occurrence of which has been increasing worldwide. Although IBD is an intensively studied human microbiome-associated disease, research on Chinese populations remains relatively limited, particularly on the mucosal microbiome. The present study aimed to analyze the changes in the mucosal microbiome associated with UC from the perspectives of medical ecology and complex network analysis.

**Results:**

In total, 56 mucosal microbiome samples were collected from 28 Chinese UC patients and their healthy family partners, followed by amplicon sequencing. Based on sequencing data, we analyzed species diversity, shared species, and inter-species interactions at the whole community, main phyla, and core/periphery species levels. We identified four opportunistic “pathogens” (i.e., *Clostridium tertium*, *Odoribacter splanchnicus*, *Ruminococcus gnavus*, and *Flavonifractor plautii*) with potential significance for the diagnosis and treatment of UC, which were inhibited in healthy individuals, but unrestricted in the UC patients. In addition, we also discovered in this study: (*i*) The positive-to-negative links (P/N) ratio, which measures the balance of species interactions or inhibition effects in microbiome networks, was significantly higher in UC patients, indicating loss of inhibition against potentially opportunistic “pathogens” associated with dysbiosis. (*ii*) Previous studies have reported conflicting evidence regarding species diversity and composition between UC patients and healthy controls. Here, significant differences were found at the major phylum and core/periphery scales, but not at the whole community level. Thus, we argue that the paradoxical results found in existing studies are due to the scale effect.

**Conclusions:**

Our results reveal changes in the ecology and network structure of the gut mucosal microbiome that might be associated with UC, and these changes might provide potential therapeutic mechanisms of UC. The four opportunistic pathogens that were identified in the present study deserve further investigation in future studies.

**Supplementary Information:**

The online version contains supplementary material available at 10.1186/s12866-021-02201-6.

## Background

Inflammatory bowel disease (IBD) exhibits chronic and relapsing inflammation of the gastrointestinal tract. Crohn’s disease (CD), ulcerative colitis (UC), and ileal CD are the three most common phenotypes of IBD. The incidence of IBD is increasing worldwide, with over 3.5 million suffers in the United States and Europe [41]. However, the pathogenesis of IBD remains unclear, although may be related to the dysregulation of the internal mucosal environment due to changes in host genes, environmental factors, gut microbes, and immune responses [1, 28, 31, 35, 42, 45]. Studies have identified that variations in several genes are associated with IBD risk, including *NOD2*, *ATG16L1*, *CARD9*, and *CLEC7A* [28], with diet, medication, and geography also involved in disease development [1, 33].

The human gut microbiome plays a key role in nutrient metabolism, pathogen protection, and immune system development. Dysbiosis of the gut microbiome is also associated with IBD [31, 48]. Common gut microbiome changes in IBD patients, for example, include a lower abundance of obligate anaerobic bacteria of short-chain fatty acids (SCFAs) and an increase in abundance of facultative anaerobes [15, 30, 42, 51, 59]. Through long-term continuous sampling of IBD patients and healthy controls, Halfvarson et al. [18] reported greater fluctuation in the gut microbiome of the IBD cohort than of healthy controls. Changes in the microbiome composition are often accompanied by gut function disorders [5, 16, 22, 35]. For example, the species and microbial metabolites associated with oxidative stress responses are significantly increased in the gastrointestinal tract of IBD patients [16]. IBD may also affect secondary bile acid metabolism in the gut microbiome [22]. Moreover, core metabolic functions are persistent and redundant across multiple gut microbial phyla, despite temporal variations in microbial taxa, genomes, and proteomes [5]. Thus, the gut microbiome appears to play a key role in the pathogenesis of IBD, although the causal relationship between microbiome dysbiosis and IBD is still unclear. Therefore, correcting the gut microbiome or its functions in patients has become a target for IBD therapy, and can be achieved through various strategies, such as antibiotics, probiotics, and fecal microbial transplantation (FMT) [11, 32, 56, 63, 64].

The objective of the current study was to investigate the influence of UC on the intestinal mucosal microbiome from the perspective of medical ecology and complex network analysis. We analyzed 56 mucosal microbiomes from 28 Chinese UC patients and their healthy family partners from three aspects, including species diversity, shared species, and inter-species relationships. All analyses were performed at the whole community, main phylum, and core/periphery species network scales.

## Materials and methods

### Study design and sample collection

All study procedures involving human subjects were approved by the Medical Ethics Board of the First People’s Hospital of Yunnan Province, China. Written and verbal informed consent were obtained from all participants. Microbial samples of intestinal mucosa were collected from 28 couples. Each couple consisted of one UC patient and a healthy control. All 56 participants were from Kunming, China, and were between the ages of 18 and 60 years old. Healthy volunteers were free of gastrointestinal illnesses and did not use drugs during endoscopy, nor did they take antibiotics during the year prior to sample collection. The diagnosis of UC was based on standard endoscopic, radiographic, and histologic criteria. All patients had been under treatment with Mesalazine. Mucosal samples were collected in the morning from the participants without undergoing bowel cleansing preparation. The intestinal mucosal sample was taken 10 cm from the anus using disposable biopsy forceps. After sampling, mucosal samples were immediately frozen in liquid nitrogen and stored at − 80 °C until DNA extraction.

### DNA extraction, 16S rRNA sequencing, and taxonomic assignment of reads

Bacterial 16S rRNA genes were amplified by polymerase chain reaction (PCR) using barcoded primers flanking the hypervariable regions V3 and V4. Amplicons were sequenced using the Illumina pyrosequencer platform. Raw data were filtered to eliminate adapter pollution and low-quality reads to obtain high-quality clean reads. The overlapping paired-end reads were then merged to tags. In total, we obtained 3,062,675 tags without primers, with 27,345 tags per sample on average. Tags were clustered into operational taxonomic units (OTUs) using scripts in USEARCH (v7.0.1090) at 97% sequence similarity [14]. OTU representative sequences were taxonomically classified using the Ribosomal Database Project (RDP) Classifier v.2.2 trained on the Greengenes database.

### Measuring microbiome diversity with Hill numbers

Microbiome diversity was quantified using Hill [24] numbers, which were reintroduced to ecology by Jost [27] and Chao et al. [8, 9], defined as:

1 $$ {}^qD={\left(\sum \limits_{i=1}^S{p}_i^q\right)}^{1/\left(1-q\right)} $$

where *D* is the diversity, *q* is the order number of diversity, *S* is the number of species (or OTUs), and *p*_*i*_ is the relative abundance of species *i*. Hill numbers at different *q* orders correspond to special ecological diversity indices, in which ^*0*^*D* is equal to species richness, ^*1*^*D* represents the exponential of the Shannon index, and ^*2*^*D* represents the reciprocal of the Simpson index. The larger the diversity order *q*, the more sensitive ^*q*^*D* is to species with high abundance.

We used effect size calculated with Cohen’s [10] *d*-statistic to examine differences in diversity between two groups. A *p*-value of < 0.05 indicated significant difference in microbiome diversity between two groups.

### Shared species analysis

The null hypothesis (H_0_) of shared species (OTUs) is that the number of shared OTUs between the two groups is no less than that between any two random groups. The alternative hypothesis (H_A_) is that the number of shared OTUs between two groups is less than that between any two random groups. We applied two algorithms to estimate the number of shared OTUs between two random groups (expected number of shared OTUs). The first algorithm (*A1*) was applied to randomly reassign OTUs and samples in the two groups, as follows: (*i*) Total number of reads (abundances) for each OTU in the two groups was first computed. (*ii*) For each OTU, the reads from the two groups were pooled together. (*iii*) The number of reads of each OTU was randomly reassigned into two new groups, and the number of shared OTUs between these two new groups was computed. The total number of reads in each new group should remain the same as that in the corresponding observed group. (*iv*) Step (*iii*) was repeated 1000 times. (*v*) The pseudo *p-*value was finally calculated, as follows:

$$ p=D/1000 $$

where *D* is the number of times the numbers of expected shared OTUs from 1000 random reassignments exceeded the number of observed shared OTUs. A *p*-value of < 0.05 indicated strong evidence to reject the null hypothesis and accept the alternative hypothesis.

The second algorithm (*A2*) was used to randomly reassign samples only, with the following steps: (*i*) All samples from the two groups were pooled together. (*ii*) The samples were randomly reassigned into two new groups. The total number of samples in the two new groups should remain the same as that in the corresponding observed groups. The number of shared OTUs between the two new groups was computed. (*iii*) Step (*ii*) was repeated 1000 times. (*iv*) The pseudo *p*-value was calculated as follows:

$$ p=D/1000 $$

where *D* is the number of times that the number of expected shared OTUs from 1000 random reassignments exceeded the number of observed shared OTUs. A *p*-value of < 0.05 indicated strong evidence to reject the null hypothesis and accept the alternative hypothesis.

### Species co-occurrence network (SCN) analysis

To reduce the noise of spurious OTUs, we filtered those OTUs with total reads from all samples of < 25. As the number of samples for each group was 28, the OTUs removed were equivalent to singletons with approximately one read per sample. Spearman’s correlation coefficients computed with the relative abundance of OTUs were adjusted with the false discovery rate (FDR) control with *p* = 0.05. Cytoscape (v2.8.3) was used to visualize networks [54] and the iGraph R-package [12] was used to compute basic network properties. The MCODE plug-in [3] of Cytoscape was used to detect network clusters (modules). In addition, we also detected the positive-to-negative links (P/N) ratios in the SCNs, as introduced by Ma [36].

### Core/periphery network (CPN) analysis

Core-periphery structures in a network consist of two classes of nodes, i.e., dense cohesive core nodes and sparse connected periphery nodes [6]. In an ideal core-periphery network, core nodes are fully connected to each other and to some periphery nodes, whereas periphery nodes are not connected with other periphery nodes [13]. The objective function (*ρ*) is used to measure how well the real structure approximates the ideal, defined as:

2 $$ \rho =\sum \limits_{i,j}{a}_{ij}{\delta}_{ij} $$

In the equation, *a*_*ij*_ represents the presence or absence of the link between node *i* and node *j*, where *a*_*ij*_ = 1 if node *i* and node *j* are linked, and 0 otherwise. *δ*_*ij*_ indicates the presence or absence of a link between node *i* and node *j* in the ideal core/periphery network, where *δ*_*ij*_ = 1 if node *i* or/and node *j* are core, and 0 otherwise. Let *A* be the adjacency matrix of *a*_*ij*_, and *∆* be the adjacency matrix of *δ*_*ij*_. When *A* and *∆* are identical, the measure *ρ* achieves its maximum value. When *ρ* is maximum, we can classify the node into either core or periphery based on *δ*. We implemented the CPN analysis in Python using code provided by Ma & Ellison [37].

## Results

### Bioinformatics analysis of sequencing data

The 28 UC patients included 11 females and 17 males, and 28 healthy controls included 17 females and 11 males. There were 842 and 860 OTUs in the healthy and UC groups, respectively. Fifteen known phyla were identified in the mucosal microbiome, with *Firmicutes*, *Bacteroidetes*, and *Proteobacteria* found to be dominant.

In addition, we built the SCNs based on the mucosal microbiomes of the healthy and UC subjects, and divided the OTUs (species) in each network into core and periphery groups using CPN analysis, which can reveal global characteristics of network structure and stability. The results are listed in Table S1. In the microbiome of healthy individuals, there were 146 core species and 170 periphery species. In the microbiome of UC patients, there were 190 core species and 206 periphery species.

### Mucosal microbiome diversity based on Hill numbers

**Fig. 1:**
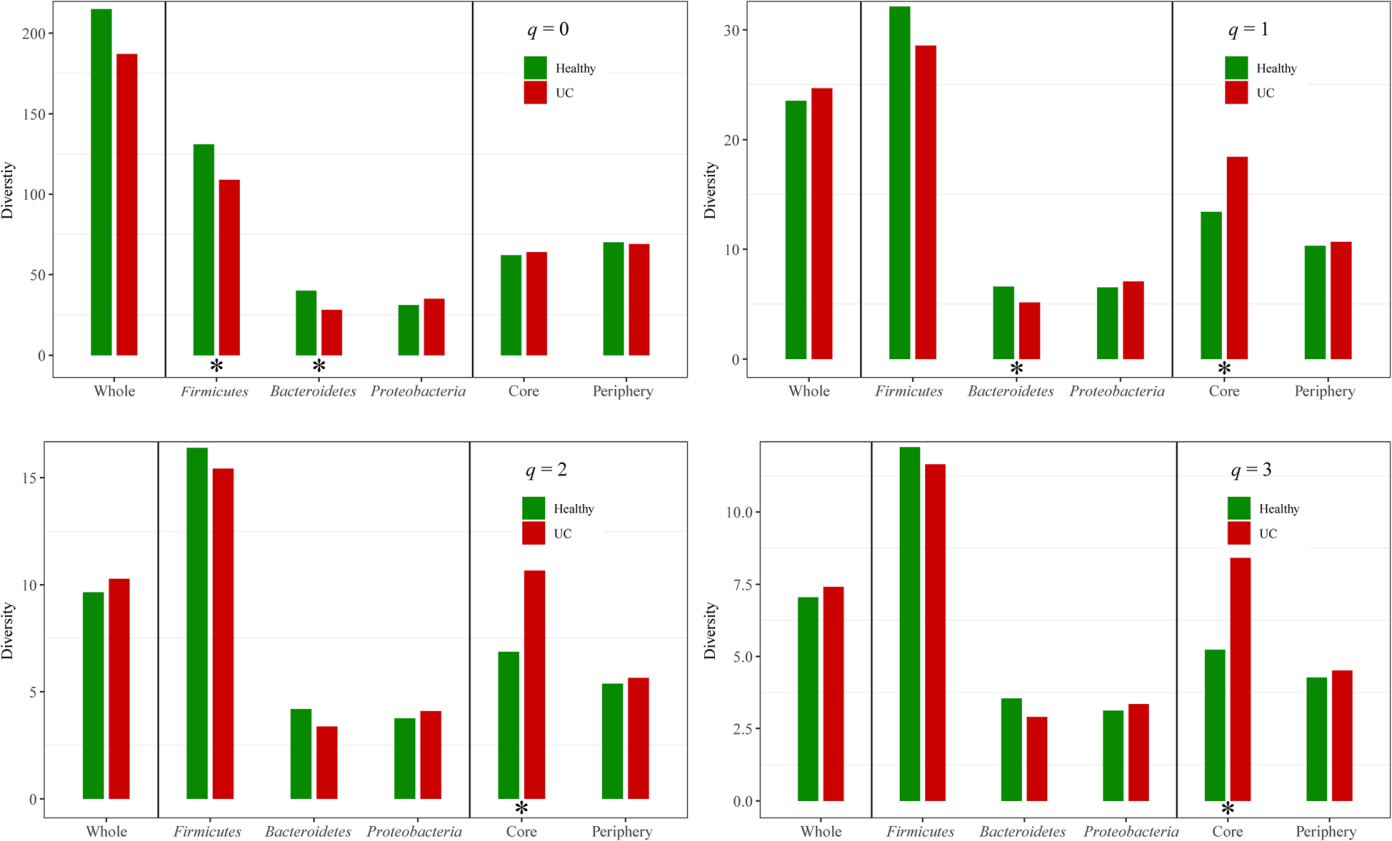
Hill numbers (diversity) of mucosal microbiome at whole community, major phylum, and core/periphery species levels. Green bar represents Hill number of healthy individuals, red bar represents Hill number of UC patients. Asterisks (*) indicate significant difference (*p* ≤ 0.05) based on Cohen’s [10] *d*-statistic

The *alpha* diversity of the mucosal microbiome was quantified using Hill numbers. For different diversity orders (*q*), the Hill number corresponds to different ecological diversity indices, including species richness, Shannon, and Simpson indices. The influence of UC on the mucosal microbiome was explored at three levels, including the whole community, main phylum, and core/periphery species scales. Figure 1 and Table S2 display the diversity of the mucosal microbiomes based on Hill numbers at four diversity orders (*q*) for healthy individuals and UC patients, as well as the significance test results.

First, the diversity of the whole mucosal microbiome was quantified with all species in the community sample. At the whole community level, there were no significant differences in the Hill numbers of the mucosal microbiomes between healthy individuals and UC patients (Table S2 and Fig. 1).

We next quantified the diversities of the three major phyla (i.e., *Firmicutes*, *Bacteroidetes*, and *Proteobacteria*) in the mucosal microbiomes, and determined the influence of UC. In general, the three most common phyla in the fecal microbiome followed the order *Firmicutes* > *Bacteroidetes* > *Proteobacteria* [45, 55]. In the present study, *Firmicutes* had the highest Hill numbers in the mucosal microbiomes of both the healthy and UC groups. In the mucosal microbiome of healthy individuals, the *Bacteroidetes* Hill number was significantly higher than that of *Proteobacteria* at the diversity order *q* = 0 (Cohen’s *d* = 0.82, *p* < 0.05). However, in the mucosal microbiome of UC patients, the *Bacteroidetes* Hill number was significantly lower than that of *Proteobacteria* at the diversity order *q* = 0 (Cohen’s *d* = − 0.73, *p* ≤ 0.05). No significant differences were observed between the *Bacteroidetes* and *Proteobacteria* Hill numbers at the other three diversity orders (*q* = 1, 2, and 3). At the diversity order *q* = 0, the *Firmicutes* Hill number in the mucosal microbiome of healthy individuals was significantly higher than that of UC patients (Table S2). At the diversity orders *q* = 0 and 1, the *Bacteroidetes* Hill number in the mucosal microbiome of healthy individuals was significantly higher than that of UC patients. There was no significant difference in the *Proteobacteria* Hill number between healthy individuals and UC patients.

Third, we quantified the diversities of core and periphery species in the mucosal microbiome. At diversity orders *q* = 1, 2, and 3, the Hill numbers of the core species in the mucosal microbiome of the UC patients were significantly higher than that of the healthy individuals, although there was no significant difference in Hill numbers at *q* = 0 between healthy and UC subjects (Fig. 1 and Table S2). There were no significant differences in the Hill numbers of periphery species between healthy individuals and UC patients. This indicated that UC mainly affected core species with high abundance.

### Shared species analysis

Shared species analysis was used to detect differences in microbial composition between different groups. Table S3 lists the results of shared species analysis based on the two algorithms [38]. For each comparison, if the observed number of shared species was significantly smaller than that expected by chance, this indicated that the shared species between this group pair were not caused by chance, and there was a significant difference between their microbial compositions. We also performed shared species analysis of the mucosal microbiome at the three levels.

**Fig. 2:**
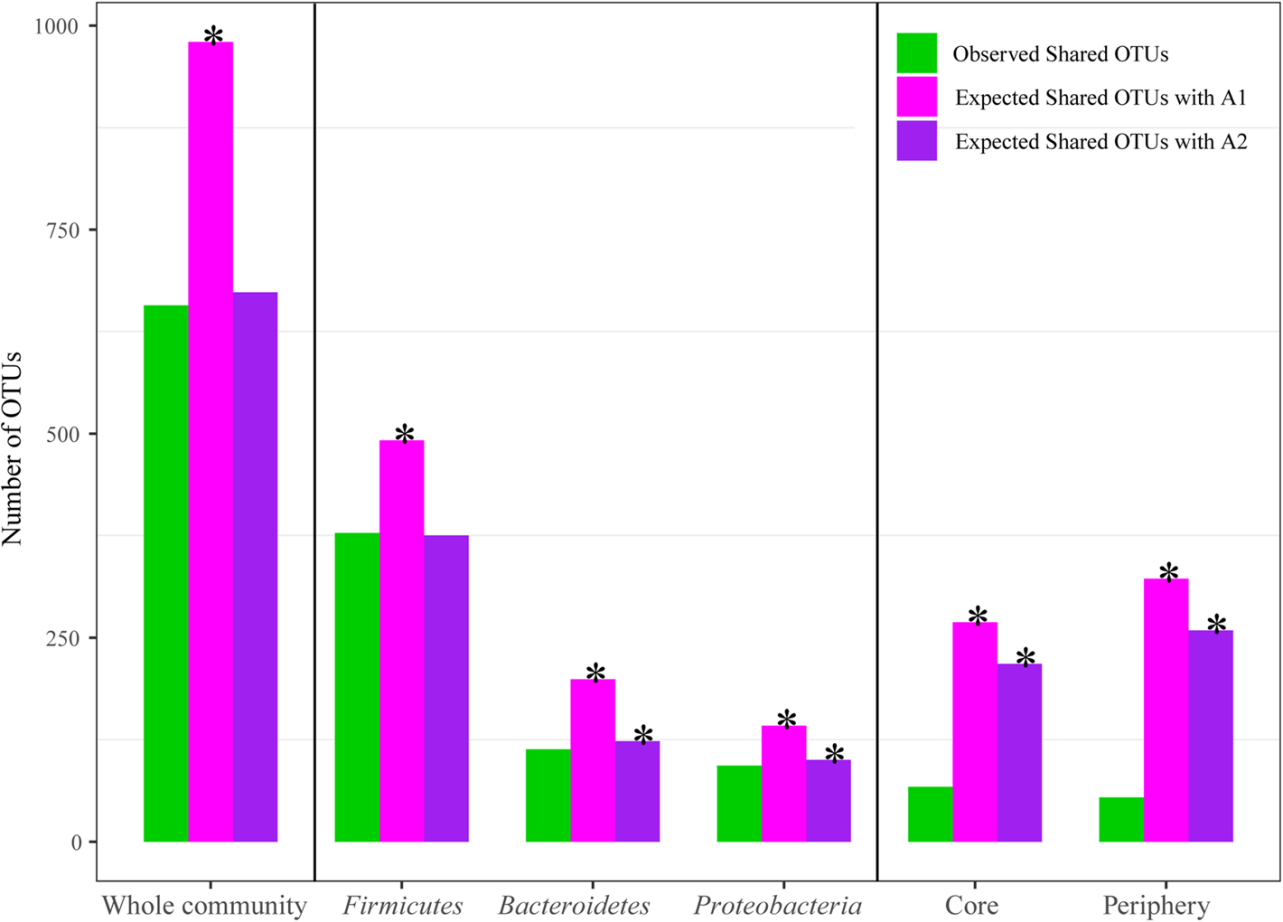
Shared mucosal microbiome species between healthy individuals and UC patients at whole community, major phylum, and core/periphery species levels. Green bar represents observed shared OTUs (species), magenta bar represents expected shared OTUs with *A1* algorithm, and purple bar represents expected shared OTUs with *A2* algorithm. Asterisks (*) indicate that number of observed shared OTUs between healthy individuals and UC patients was significantly smaller than expected by chance (*p ≤* 0.05)

With the *A1* algorithm (reshuffling reads), the observed number of shared species between the group pairs was significantly smaller than that expected by chance for all comparisons (Fig. 2 and Table S3). Compared with the *A1* algorithm, the *A2* algorithm (reshuffling samples) was more conservative. In the whole mucosal microbiome, there was no significant difference in the observed number of shared species between the healthy and UC groups versus that expected by chance. For shared species analysis of the three main phyla, *Firmicutes* showed a similar result to that found at the whole community level. However, the observed number of shared *Bacteroidetes* and *Proteobacteria* species between healthy and UC subjects was significantly smaller than that expected by chance. Furthermore, the observed number of shared core/periphery OTUs was significantly smaller than that expected by chance. As shown in Table S4, the SCNs of the healthy and UC groups shared 67 core species and 54 periphery species. Table S5 shows the shared and specific core/periphery species of each group.

Based on the diversity and shared species results, UC appeared to have little effect on the mucosa microbiome at the whole community level. However, we found that UC was associated with dysbiosis of the mucosa microbiome, characterized by changes in the main bacterial phyla, including a decrease in *Firmicutes* richness, change in *Proteobacteria* species composition, and variation in both *Bacteroidetes* diversity and species composition. In addition, UC was also associated with changes in core and periphery species in the mucosal microbiome network. According to the Vellend-Hanson synthesis [21, 61], CPN analysis can effectively detect inequalities from a node perspective, which are caused by the selection effects of the mucosa or host environment [34, 39]. Thus, the significant differences in the diversity and composition of the core/periphery species indicated that UC may influence the selection effects of the host environment on the OTUs in the mucosal microbiome.

### Species co-occurrence network (SCN) analysis

**Fig. 3:**
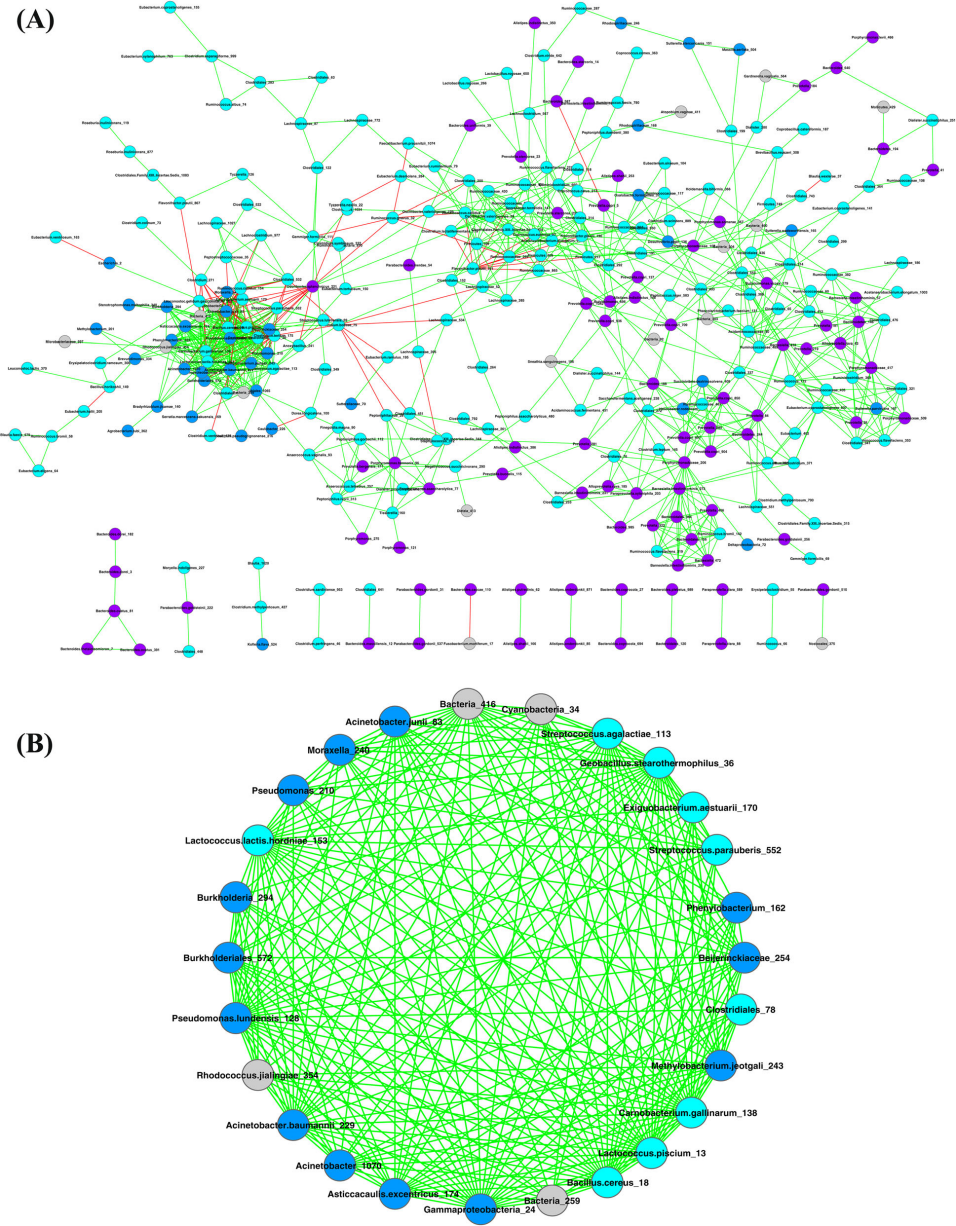
Mucosa microbiome SCN of healthy group and strongest cluster (score > 5): **a** Mucosa microbiome SCN of healthy group; **b** Strongest cluster (#1 cluster). Nodes in cyan, OTUs of *Firmicutes* phylum; nodes in blue, OTUs of *Proteobacteria* phylum; nodes in purple, OTUs of *Bacteroidetes* phylum; nodes in gray, OTUs of other phyla; edges in green, positive correlations; edges in red, negative correlations

**Fig. 4:**
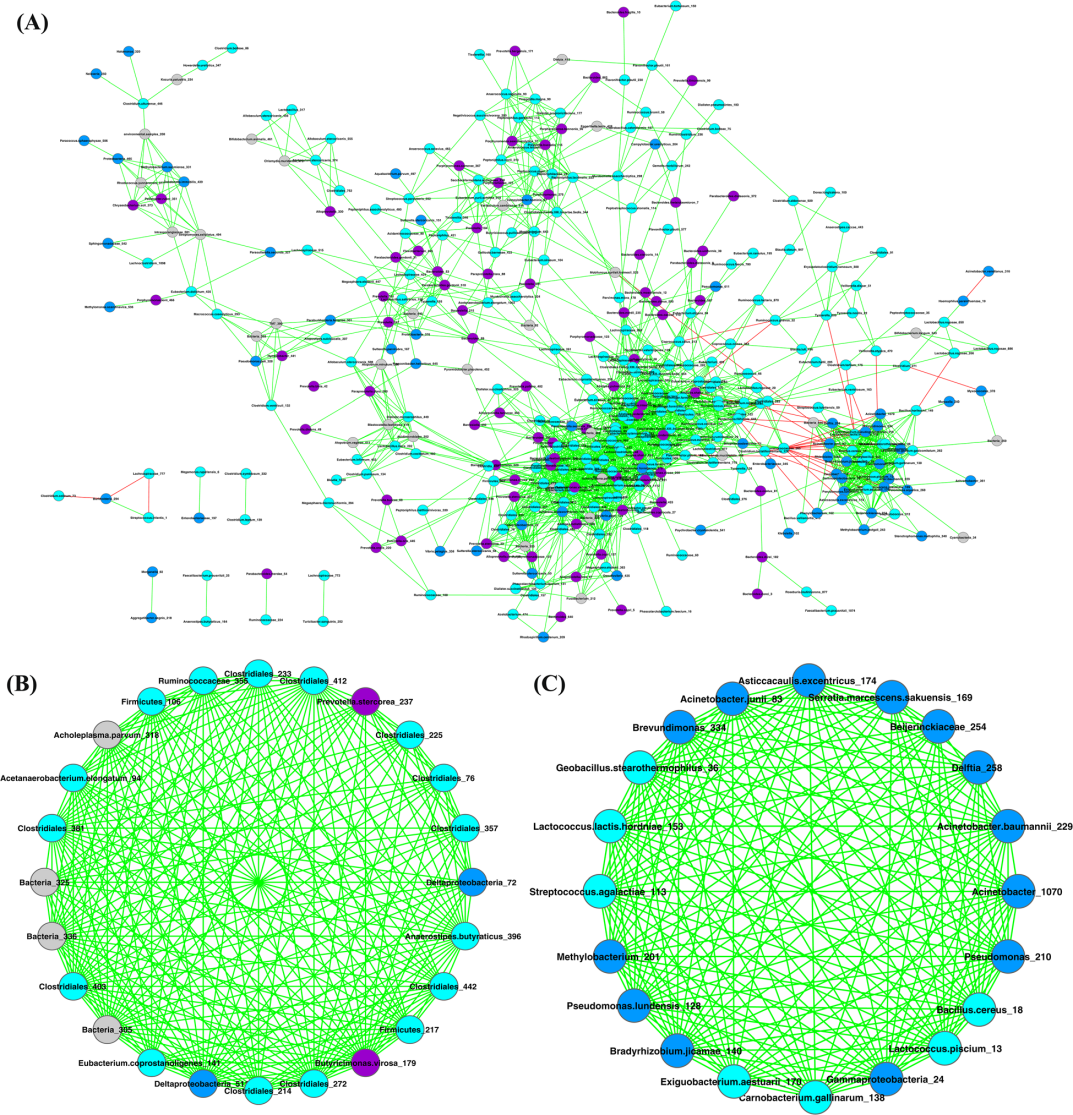
Mucosa microbiome SCN of UC group and top two strongest clusters (scores > 5): **a** Mucosa microbiome SCN of UC group; **b** strongest cluster (#1 cluster); **c** second strongest cluster (#2 cluster). Nodes in cyan, OTUs of *Firmicutes* phylum; nodes in blue, OTUs of *Proteobacteria* phylum; nodes in purple, OTUs of *Bacteroidetes* phylum; nodes in gray, OTUs of other phyla; edges in green, positive correlations; edges in red, negative correlations

We first removed relatively sparse OTUs from the two mucosal microbiomes (as described in the Materials and Methods). The mucosal microbiome SCNs of the healthy (healthy-SCN) and UC (UC-SCN) groups were then constructed, as illustrated in Figs. 3 and 4, respectively. Table S6 shows the basic network properties of the two SCNs. The number of nodes in the two SCNs was similar, but the number of edges in the UC-SCN was two times that in the healthy-SCN, which resulted in a higher average degree. The number of connected components in the healthy-SCN was nearly twice that of the UC-SCN, indicating that the fragmentation degree of the healthy-SCN was higher than that of the UC-SCN.

The MCODE algorithm was used to detect network clusters (modules). Results are shown in Table S7, and include the cluster number, cluster score, number of nodes, and number of edges for each cluster. The cluster score is a measure of cluster density, with a higher cluster score indicating a stronger corresponding cluster. The healthy-SCN had only one strong cluster (#1 cluster, score > 5), whereas the UC-SCN had two strong clusters (#1 and #2 clusters, scores > 5). These three clusters consisted of species from the phyla *Firmicutes* and *Proteobacteria*. As shown in Fig. 3b, 85% (22/26) of OTUs (nodes) in the #1 cluster of the healthy-SCN belonged to *Firmicutes* and *Proteobacteria*. As shown in Fig. 4b and c, in the UC-SCN, 75% (18/24) of OTUs in the #1 cluster and all OTUs in the #2 cluster were from *Firmicutes* and *Proteobacteria*. *Bacteroidetes* species were scattered in both SCNs without forming a strong cluster. The healthy-SCN #1 cluster and UC-SCN #2 cluster had similar species composition and network structure. For species composition, 15 OTUs were shared between the two clusters (see Table S8). For network structure, all interactions within the two clusters were positive/cooperative, and these clusters both contained more than half of the negative/inhibitive interactions of their SCNs (Figures S1 and S2). Thus, these two clusters may play a similar role in their own mucosal microbiome.

In addition, based on the P/N ratios in the SCNs, UC was associated with a loss in mucosal microbiome balance in patients. As shown in Table S9, the P/N ratio in the UC-SCN was five times higher than that in the healthy-SCN, indicating that the number of inhibitive interactions decreased in the mucosa microbiome of UC patients. The negative links were mainly found within the three main phyla, i.e., *Firmicutes*, *Bacteroidetes*, and *Proteobacteria*. In the healthy-SCN, 59% (45/76) of negative links were found within *Firmicutes* and 22% (17/76) were found between *Firmicutes* and *Proteobacteria*. In the UC-SCN, the number of negative links decreased to less than half that in the healthy-SCN, which was mainly due to the considerable reduction of negative links within *Firmicutes* and between *Firmicutes* and *Bacteroidetes*. Compared with the healthy-SCN, we identified a marked increase in the P/N ratios within and among the three main phyla in the UC-SCN. As mentioned above, we used CPN analysis to divide the OTUs in the network into two groups, i.e., core and periphery. In both the healthy- and UC-SCNs, more than 50% of positive and negative links were found within the core species. Similar to the patterns observed at the whole community and main phylum scales, the core, periphery, and core-periphery species in the UC-SCN had higher P/N ratios than those in the healthy-SCN.

### Potential “pathogens” or research targets of UC

The five times higher P/N ratio in UC patients signaled loss of inhibition to certain potentially opportunistic pathogens. Based on the changes in abundance and P/N ratio of each OTU in UC patients, we set the following selection criteria for potential pathogens: (*i*) significant differences in abundance between the healthy and UC groups (Wilcoxon test *p ≤* 0.05); (*ii*) five or more negative links and a P/N ratio of < 0.5 in the healthy-SCN; but (*iii*) a significantly higher P/N ratio in the UC-SCN.

In total, four OTUs or species met the selection criteria, including *Clostridium tertium* (OTU ID: 175), *Odoribacter splanchnicus* (OTU ID: 221), *Ruminococcus gnavus* (OTU ID: 32), and *Flavonifractor plautii* (OTU ID: 161) (see Table S10 for details). Results showed an increase in *C. tertium*, *R. gnavus*, and *F. plautii*, but a decrease in *O. splanchnicus* in the mucosal microbiome of UC patients. In addition, *C. tertium* had the greatest number of negative links in the healthy individuals, but only one negative link in the UC patients. Both *O. splanchnicus* and *F. plautii* had 11 and nine negative links, respectively, in healthy individuals, but none in UC patients. In the healthy-SCN, *R. gnavus* only had negative links (no positive links), whereas, in the UC-SCN, *R. gnavus* had 1.5 times more positive links than negative links. Furthermore, *C. tertium* and *O. splanchnicus* were core species in both healthy and UC subjects, whereas, *R. gnavus* and *F. plautii* were core species in healthy individuals but periphery species in UC subjects.

## Discussion and conclusions

We compared mucosal microbiome samples from 28 UC patients and their healthy partners. Analyses focused on species diversity, shared species, and species interactions at three scales (i.e., whole community, major phylum, and core/periphery critical network). Our study revealed three major findings, as summarized below.

First, the differences between UC patients and healthy controls in diversity and shared species were scale dependent. At the whole community scale, the differences in species diversity and composition were not obvious. However, at the major phylum and core/periphery species scales, significant differences were found between the UC patients and healthy controls. Although a reduction in gut microbial diversity in UC patients has been reported in multiple studies [31, 43, 46, 65], other research has found there to be no significant differences between UC patients and healthy individuals [7, 20, 52]. These inconsistencies are likely related to differences in study design, subject status, and sample collection processes. Compared with the whole gut mucosal microbiome, compositional alterations at the phylum level are more consistent across different studies, e.g., decrease in *Firmicutes* and *Bacteroidetes* [31, 45] and increase in *Proteobacteria* [16, 44]. Moreover, the compositional variation at taxonomic scales plays an important role in explaining pathogenesis and assessing therapeutic efficacy. For example, Ishikawa et al. [25, 26] found that FMT following antibiotic pretreatment can effectively improve gut dysbiosis caused by loss of *Bacteroidetes* in UC patients. The significance of our first finding reinforces the critical importance of scale in studies on microbiome-associated diseases, and provides the core/periphery network structure as a new scale for microbiome research.

Second, it is well recognized that UC is associated with dysbiosis or loss of balance in the gut microbiome of patients [11, 31, 44, 46, 51]. However, no existing studies have offered quantitative measures to demonstrate the effects of dysbiosis. In other words, the characterization of dysbiosis is often presented in the form of qualitative description. Our study revealed a considerably higher (five times) P/N ratio in UC patients, signaling the loss of inhibition to certain potentially opportunistic pathogens. It should be noted that we use the term “pathogen” somewhat differently from the traditional Koch’s postulates, i.e., certain bacterial species are responsible for disease. Current research on microbiome-associated diseases still cannot, in many cases, answer the simple question—what are the disease pathogens based on the traditional Koch’s postulates?

Third, the considerable change in P/N ratio not only quantified the effects of dysbiosis but also helped to detect the “culprits”. Based on the variations in composition and P/N ratio, we identified four potentially opportunistic pathogens, i.e., *C. tertium*, *O. splanchnicus*, *R. gnavus*, and *F. plautii*. *Clostridium tertium* is an anaerobic gram-positive bacterium in the phylum *Firmicutes*. Although *C. tertium* has traditionally been considered non-pathogenic, various studies have reported it to be a significant cause of bacteremia and other infections in neutropenia patients [29, 49, 50, 53, 60]. In addition, *C. tertium* infections in non-neutropenic patients have also been reported recently [57, 58, 62]. *Odoribacter splanchnicus* (*Bacteroides splanchnicus*) is an anaerobic gram-negative bacterium in *Bacteroidetes*. Although it is found in normal human colonic microbiomes, it has the potential to be an opportunistic pathogen [17]. In the present study, we found reduced abundance of *O. splanchnicus* in UC patients, consistent with that reported in CD patients [42]. *Odoribacter splanchnicus* can produce acetate, propionate, and butyrate, which are associated with the biosynthesis of SCFAs [17]. The effect of *O. splanchnicus* variation on UC patients may also be related to the reduction in SCFAs [17, 42]. *Ruminococcus gnavus* is an anaerobic gram-positive bacterium in the phylum *Firmicutes*. An increase in *R. gnavus* has been reported previously in the gut microbiome of IBD patients [19, 46, 65], as found in the present study. Henke et al. [23] recently reported that *R. gnavus* can produce an inflammatory polysaccharide, revealing a possible relationship between *R. gnavus* and IBD pathogenesis. *Flavonifractor* (*Eubacterium*) *plautii* is another anaerobic gram-positive member of *Firmicutes*. Compared with healthy individuals, patients with IBD have significantly higher concentrations of mucosal IgG in their gut, although corresponding antigens remain unclear [4, 40]. Recent studies have found that *F. plautii* is associated with enhancement of host intestinal IgG levels in IBD patients [2, 47]. In summary, our findings reinforce the potential importance of these four species, which deserve further investigation in future studies.

## Supplementary Information

**Additional file 1.**

## Data Availability

Sequence data from this study were deposited in the GenBank Sequence Read Archive under accession SRA BioProject PRJNA600852.
