# Supplementary material for: Ecological and network analyses identify four microbial species with potential significance for the diagnosis/treatment of ulcerative colitis (UC)
Source: BMC Microbiol. 2021 May 4;21:138. doi: 10.1186/s12866-021-02201-6 (PMC8097971; doi:10.1186/s12866-021-02201-6)
Supplement: Supplementary file 1 — Additional file 1. [file 12866_2021_2201_MOESM1_ESM.doc]

**Ecological and network analyses identify four microbial species with potential significance for the diagnosis/treatment of ulcerative colitis (UC)**

Wendy Li ^1,2*^ Yang Sun ^3*^ Lin Dai^4^ Hongju Chen^1,2,5^ Bin Yi^5^  Junkun Niu^3^

Lan Wang^3^  Fengrui Zhang^3^ Juan Luo^3^ Kunhua Wang^6^ Lianwei Li^1,2^ Quan Zou^7^

ZhanShan (Sam) Ma^1,2,8#^  Yinglei Miao^3#^

^1^Computational Biology and Medical Ecology Lab, State Key Laboratory of Genetic Resources and Evolution, Kunming Institute of Zoology, Chinese Academy of Sciences, China

^2^Kunming College of Life Sciences, University of Chinese Academy of Sciences, China

^3^Department of Gastroenterology, The First Affiliated Hospital of Kunming Medical University, Yunnan Institute of Digestive Disease, Kunming, Yunnan, China.

^4^Faculty of Science, Kunming University of Science and Technology, Kunming, China

^5^College of Mathematics, Honghe University, Mengzi, Yunnan Province, China

^6^Department of General Surgery, The First Affiliated Hospital of Kunming Medical University, Yunnan Institute of Digestive Disease, Kunming, Yunnan, China.

^7^Institute of Fundamental and Frontier Sciences, University of Electronic Science and Technology of China, Chengdu, China

^8^Center for Excellence in Animal Evolution and Genetics, Chinese Academy of Sciences, China

#Co-Correspondence:

Address correspondence to Yinglei Miao: [miaoyinglei@yeah.net](mailto:miaoyinglei@yeah.net)

Address correspondence to Zhanshan (Sam) Ma: [ma@vandals.uidaho.edu](mailto:ma@vandals.uidaho.edu)

^*^ These authors contributed equally to this work: Wendy Li, Yang Sun

**Online Supplementary Figures**


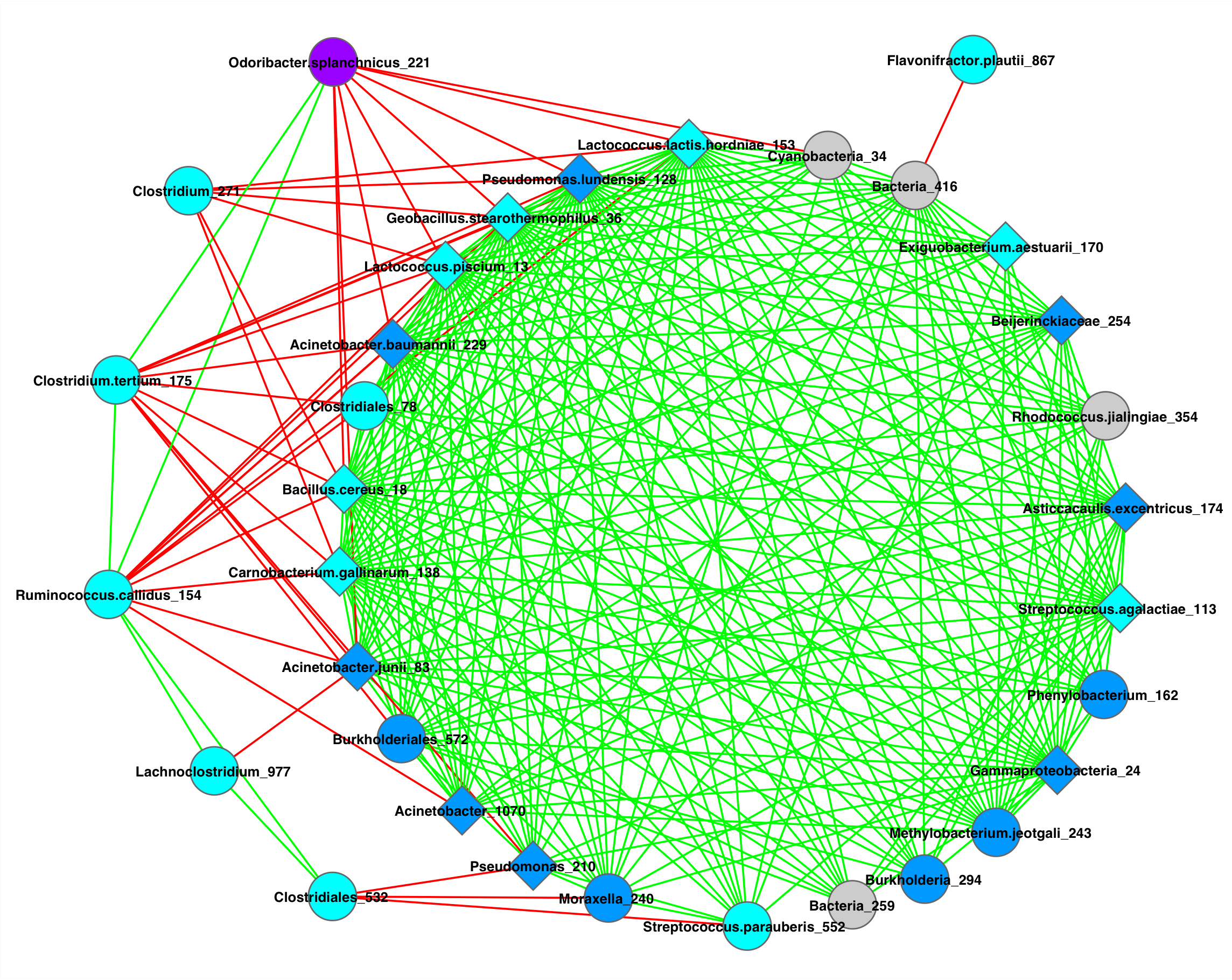


**Fig S1.** The #1 cluster and the negative relationships with it in the healthy-SCN: The circle of OTUs in the center of the figure is the #1 cluster of the healthy-SCN. The OTUs that are negatively connected to this circle were not belonging to the #1 cluster, but to the “enemies” of this cluster in the SCN. Nodes in diamond—the 15 OTUs that shared between the #1 cluster of the healthy-SCN and the #2 cluster of the UC-SCN, nodes in cyan—the OTUs of *Firmicutes* phylum, nodes in blue—the OTUs of *Proteobactera* phylum, nodes in purple— the OTUs of *Bacteroidetes* phylum, nodes in gray—the OTUs of other phyla; edges in green— positive correlations, edges in red—negative correlations.


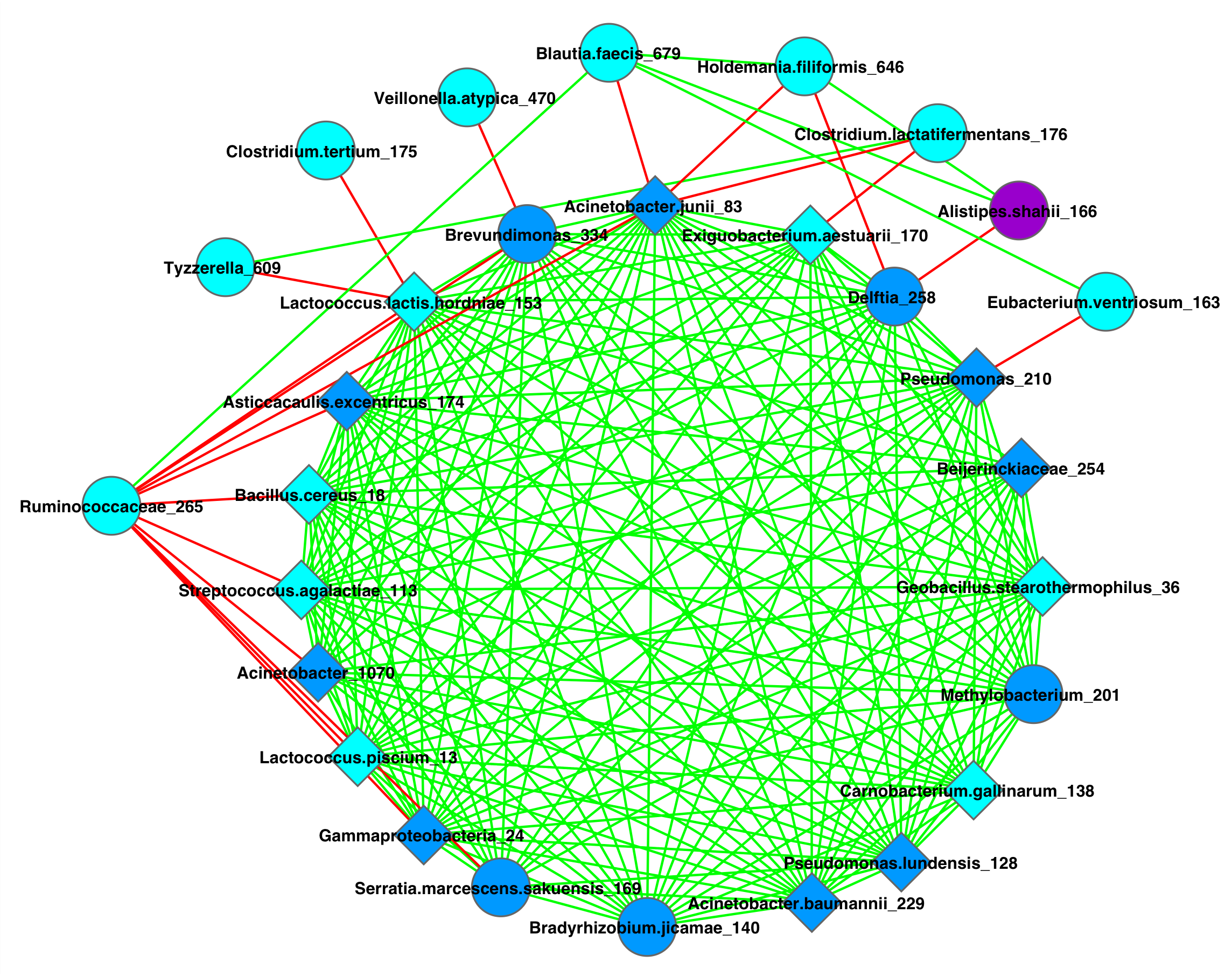


**Fig S2.** The #2 cluster and the negative relationships with it in the UC-SCN: The circle of OTUs in the center of the figure is the #2 cluster of the UC-SCN. The OTUs that are negatively connected to this circle were not belonging to the #2 cluster, but to the “enemies” of this cluster in the SCN. Nodes in diamond—the 15 OTUs that shared between the #1 cluster of the healthy-SCN and the #2 cluster of the UC-SCN, nodes in cyan—the OTUs of *Firmicutes* phylum, nodes in blue—the OTUs of *Proteobactera* phylum, nodes in purple— the OTUs of *Bacteroidetes* phylum, nodes in gray—the OTUs of other phyla; edges in green— positive correlations, edges in red—negative correlations.

**Online Supplementary Tables**

**Table S1**. The core/periphery and nested structures in the species co-occurrence networks (SCNs)

of the mucosal microbiomes of the healthy and UC groups

| **Treatments** | ***ρ*** | **Ratio of C/P** | **Density Matrix** | | | **Nested-ness**  **(*S*)** |
| --- | --- | --- | --- | --- | --- | --- |
|  |  |  | B11 | B12(21) | B22 |  |
| Healthy | 0.119 | 0.462 | 0.052 | 0.012 | 0.011 | 0.035 |
| UC | 0.145 | 0.480 | 0.070 | 0.015 | 0.013 | 0.047 |

**Table S2. Mean and standard error of Hill numbers (diversity) of mucosal microbiome at whole community, major phylum, and core/periphery species levels, and results (*d*-statistic and *p*-value) for effect-size test on diversity (highlighted cells show significant differences at *p ≤* 0.05)**

| **Treatment** | | **Statistic** | ***q* = 0** | ***q* = 1** | ***q* = 2** | ***q* = 3** |
| --- | --- | --- | --- | --- | --- | --- |
| Whole community | Healthy | Mean | 215 | 23.530 | 9.650 | 7.051 |
|  |  | Std. Err. | 56.883 | 10.855 | 4.319 | 2.993 |
|  | UC | Mean | 187 | 24.676 | 10.279 | 7.416 |
|  |  | Std. Err. | 64.104 | 11.825 | 4.983 | 3.307 |
|  | Healthy vs. UC | Cohen’s d | 0.510 | -0.090 | -0.140 | -0.120 |
|  |  | *p*-value of d | 0.060 | 0.730 | 0.610 | 0.640 |
| **Phylum** | | | | | | |
| *Firmicutes* | Healthy | Mean | 131 | 32.136 | 16.393 | 12.247 |
|  |  | Std. Err. | 41.761 | 14.604 | 8.742 | 6.613 |
|  | UC | Mean | 109 | 28.563 | 15.422 | 11.656 |
|  |  | Std. Err. | 44.844 | 15.867 | 9.162 | 7.071 |
|  | Healthy vs. UC | Cohen’s d | 0.550 | 0.290 | 0.140 | 0.110 |
|  |  | *p*-value of d | 0.050 | 0.290 | 0.610 | 0.680 |
| *Bacteroidetes* | Healthy | Mean | 40 | 6.595 | 4.189 | 3.543 |
|  |  | Std. Err. | 11.455 | 2.746 | 1.765 | 1.471 |
|  | UC | Mean | 28 | 5.143 | 3.374 | 2.902 |
|  |  | Std. Err. | 13.433 | 2.826 | 1.718 | 1.397 |
|  | Healthy vs. UC | Cohen’s d | 1.000 | 0.560 | 0.490 | 0.460 |
|  |  | *p*-value of d | 0.000 | 0.050 | 0.080 | 0.090 |
| *Proteobacteria* | Healthy | Mean | 31 | 6.516 | 3.759 | 3.119 |
|  |  | Std. Err. | 8.118 | 4.164 | 2.494 | 1.984 |
|  | UC | Mean | 35 | 7.062 | 4.095 | 3.346 |
|  |  | Std. Err. | 9.223 | 4.919 | 2.887 | 2.231 |
|  | Healthy vs. UC | Cohen’s d | -0.450 | -0.070 | -0.100 | -0.090 |
|  |  | *p*-value of d | 0.100 | 0.790 | 0.720 | 0.740 |
| **Core/Periphery** | | | | | | |
| Core | Healthy | Mean | 62 | 13.413 | 6.868 | 5.230 |
|  |  | Std. Err. | 17.580 | 7.786 | 4.459 | 3.126 |
|  | UC | Mean | 64 | 18.435 | 10.669 | 8.419 |
|  |  | Std. Err. | 23.965 | 10.468 | 6.460 | 5.075 |
|  | Healthy vs. UC | Cohen’s d | -0.090 | -0.570 | -0.740 | -0.810 |
|  |  | *p*-value of d | 0.740 | 0.040 | 0.010 | 0.010 |
| Periphery | Healthy | Mean | 70 | 10.320 | 5.376 | 4.269 |
|  |  | Std. Err. | 19.381 | 6.054 | 3.258 | 2.403 |
|  | UC | Mean | 69 | 10.674 | 5.646 | 4.510 |
|  |  | Std. Err. | 19.359 | 5.246 | 3.222 | 2.600 |
|  | Healthy vs. UC | Cohen’s d | 0.050 | -0.100 | -0.110 | -0.110 |
|  |  | *p*-value of d | 0.850 | 0.720 | 0.680 | 0.670 |

**Table S3***.* Results of shared species between each pair-wise group (highlighted cells show significant differences at *p* ≤ 0.05)

| **Treatment** | **Observed shared OTUs** | **Algorithm A1** | | | **Algorithm A2** | | |
| --- | --- | --- | --- | --- | --- | --- | --- |
|  |  | **Expected shared OTUs** | **O/E (%)** | ***p-*value** | **Expected shared OTUs** | **O/E (%)** | ***p-*value** |
| Whole community | 657 | 980 | 67.045 | 0.000 | 673 | 97.581 | 0.118 |
| **Phylum** | | | | | | | |
| *Firmicutes* | 378 | 492 | 76.8 | 0.000 | 375 | 100.8 | 0.576 |
| *Bacteroidetes* | 113 | 199 | 56.7 | 0.000 | 123 | 92.1 | 0.011 |
| *Proteobacteria* | 93 | 142 | 65.5 | 0.000 | 100 | 92.9 | 0.017 |
| **Core/Periphery** | | | | | | | |
| Core | 67 | 269 | 24.9 | 0.000 | 218 | 30.8 | 0.000 |
| Periphery | 54 | 322 | 16.8 | 0.000 | 259 | 20.9 | 0.000 |

**Table S4**. Number of shared and specific core/periphery OTUs between healthy and UC SCNs

| **Treatment** | **Num. of shared OTUs** | **Num. of specific OTUs** | | **% of shared OTUs** | | |
| --- | --- | --- | --- | --- | --- | --- |
|  |  | Healthy | UC | Healthy | | UC |
| Core | 67 | 79 | 123 | 22.9 | 17.6 | |
| Periphery | 54 | 116 | 152 | 15.9 | 13.1 | |

**Table S5**. The shared and specific core/periphery species between the healthy and UC groups

| **Core** | | |
| --- | --- | --- |
| **Shared Cores** | **Specific Cores** | |
|  | **Healthy** | **UC** |
| *Bacteria_416* | *Ruminococcaceae_489* | *Ruminococcaceae_964* |
| *Clostridiales_412* | *Clostridiales_522* | *Acholeplasma.parvum_318* |
| *Streptococcus.parauberis_552* | *Ruminococcus.flavefaciens_319* | *Staphylococcus_312* |
| *Oxalobacter.formigenes_379* | *Clostridiales_314* | *Peptoniphilus.ivorii_313* |
| *Oscillibacter.valericigenes_29* | *Porphyromonadaceae_417* | *Clostridiales.Family.XIII..Incertae.Sedis_315* |
| *Gammaproteobacteria_24* | *Clostridiales_553* | *Lachnospiraceae_551* |
| *Bacteroides.coprocola_27* | *Clostridiales_550* | *Blautia.faecis_679* |
| *Asticcacaulis.excentricus_174* | *Lachnospiraceae_772* | *Sutterella.stercoricanis_50* |
| *Clostridium.tertium_175* | *Clostridiales_388* | *Sutterella.stercoricanis_56* |
| *Exiguobacterium.aestuarii_170* | *Lachnospiraceae_52* | *Alistipes.shahii_253* |
| *Prevotella.bergensis_171* | *Barnesiella.intestinihominis_57* | *Anaerostipes.butyraticus_396* |
| *Butyricimonas.virosa_179* | *Beijerinckiaceae_254* | *Faecalibacterium.prausnitzii_25* |
| *Clostridiales_436* | *Anaerococcus.tetradius_257* | *Eubacterium.rectale_21* |
| *Ruminococcaceae_430* | *Parabacteroides.goldsteinii_256* | *Dialister.propionicifaciens_177* |
| *Barnesiella_433* | *Bacteria_259* | *Megamonas.funiformis_1112* |
| *Acinetobacter_1070* | *Prevotella.copri_709* | *Clostridiales.Family.XIII..Incertae.Sedis_1083* |
| *Acinetobacter.baumannii_229* | *Peptoniphilaceae_28* | *Alistipes.onderdonkii_871* |
| *Odoribacter.splanchnicus_221* | *Acidaminococcus.fermentans_431* | *Pelomonas.aquatica_268* |
| *Pseudomonas.lundensis_128* | *Mollicutes_429* | *Ruminococcus.faecis_780* |
| *Clostridiales_122* | *Rhodococcus.jialingiae_354* | *Intrasporangiaceae_581* |
| *Ruminococcaceae_60* | *Tyzzerella_126* | *Lachnospiraceae_586* |
| *Clostridiales_65* | *Ruminococcaceae_463* | *Clostridiales_451* |
| *Porphyromonas.levii_466* | *Bacteria_304* | *Barnesiella_450* |
| *Ruminococcaceae_269* | *Brevibacillus.reuszeri_308* | *Porphyromonadaceae_457* |
| *Lachnospiraceae_261* | *Dialister_280* | *Clostridiales.Family.XIII..Incertae.Sedis_454* |
| *Leuconostoc.gelidum.gasicomitatum_262* | *Prevotella_289* | *Clostridiales_225* |
| *Ruminococcaceae_117* | *Cyanobacteria_34* | *Caulobacter_226* |
| *Streptococcus.agalactiae_113* | *Peptostreptococcaceae_35* | *Deltaproteobacteria_511* |
| *Gemmiger.formicilis_111* | *Ruminococcus.gnavus_32* | *Ruminococcaceae_355* |
| *Geobacillus.stearothermophilus_36* | *Phenylobacterium_162* | *Clostridiales_357* |
| *Sutterella.parvirubra_167* | *Flavonifractor.plautii_161* | *Firmicutes_352* |
| *Acinetobacter.junii_83* | *Prevotella_84* | *Holdemania.filiformis_646* |
| *Rhizobiales_1065* | *Ruminococcaceae_426* | *Mogibacterium_643* |
| *Bacteroides_640* | *Eubacterium.coprostanoligenes_507* | *Porphyromonas_121* |
| *Stenotrophomonas.maltophilia_340* | *Porphyromonadaceae_509* | *Clostridiales_63* |
| *Clostridiales_152* | *Peptoniphilus.asaccharolyticus_480* | *Alistipes.putredinis_62* |
| *Lactococcus.lactis.hordniae_153* | *Clostridiales_792* | *Eubacterium.eligens_64* |
| *Ruminococcus.callidus_154* | *Bacteroidales_156* | *Campylobacter.hominis_67* |
| *Clostridiales_233* | *Porphyromonas.asaccharolytica_77* | *Ruminococcus_66* |
| *Saccharofermentans.acetigenes_238* | *Clostridium.bolteae_75* | *Gemmiger.formicilis_69* |
| *Ruminococcaceae_382* | *Clostridiales_78* | *Prevotella.buccae_68* |
| *Eubacterium.hallii_205* | *Clostridium.lactatifermentans_779* | *Lachnoclostridium_1098* |
| *Paraprevotella.xylaniphila_203* | *Bacteroidales_384* | *Bacteria_460* |
| *Clostridiales_200* | *Burkholderiales_572* | *Ruminiclostridium_545* |
| *Firmicutes_109* | *Porphyromonadaceae_206* | *Tyzzerella_609* |
| *Flavonifractor.plautii_190* | *Clostridium_271* | *Eubacterium.xylanophilum_763* |
| *Clostridiales_191* | *Ruminococcaceae_108* | *Bacteria_305* |
| *Acetanaerobacterium.elongatum_94* | *Burkholderia_294* | *Ruminococcaceae_265* |
| *Coprococcus.eutactus_97* | *Negativicoccus.succinicivorans_290* | *Butyricimonas.virosa_260* |
| *Clostridiales_403* | *Clostridiales_292* | *Ruminococcus.bromii_116* |
| *Sporobacter.termitidis_147* | *Bacteroidetes_194* | *Prevotella.buccalis_115* |
| *Methylobacterium.jeotgali_243* | *Eubacterium.ramulus_195* | *Bacteroides.caccae_110* |
| *Firmicutes_217* | *Clostridiales_532* | *Clostridiales_283* |
| *Pseudomonas_210* | *Clostridiales_321* | *Eubacterium.ventriosum_163* |
| *Porphyromonas.somerae_367* | *Prevotella_322* | *Firmicutes_309* |
| *Carnobacterium.gallinarum_138* | *Ruminococcus.bromii_142* | *Sutterella.wadsworthensis_165* |
| *Lactococcus.piscium_13* | *Dialister.succinatiphilus_144* | *Clostridium.asparagiforme_999* |
| *Clostridiales_214* | *Bacteroidetes_146* | *Morganella_82* |
| *Desulfovibrio.piger_136* | *Moraxella_240* | *Alistipes.onderdonkii_85* |
| *Porphyromonas.bennonis_96* | *Ruminiclostridium_660* | *Ruminococcaceae_500* |
| *Serratia.marcescens.sakuensis_169* | *Ruminococcaceae_865* | *Desulfovibrio_425* |
| *Flavonifractor.plautii_867* | *Gardnerella.vaginalis_564* | *Bacteria_336* |
| *Bacillus.cereus_18* | *Prevotella_219* | *Brevundimonas_334* |
| *Lachnospiraceae_87* | *Helicobacter.rodentium_130* | *Barnesiella.intestinihominis_335* |
| *Anoxybacillus_241* | *Nostocales_375* | *Franconibacter.helveticus_645* |
| *Prevotella_184* | *Bacteroides_188* | *Clostridiales_349* |
| *Clostridium.scindens_889* | *Barnesiella_472* | *Tissierellia_346* |
|  | *Ruminococcaceae_405* | *Clostridiales.Family.XIII..Incertae.Sedis_344* |
|  | *Ruminococcus.callidus_189* | *Eubacterium.coprostanoligenes_155* |
|  | *Prevotella_41* | *Deltaproteobacteria_72* |
|  | *Peptoniphilus.duerdenii_380* | *Eubacterium.ruminantium_79* |
|  | *Bacteroides.coprocola_694* | *Flavonifractor.plautii_230* |
|  | *Roseburia.inulinivorans_119* | *Myxococcales_378* |
|  | *Coprococcus.comes_363* | *Alistipes.shahii_166* |
|  | *Lachnoclostridium_567* | *Lachnospiraceae_385* |
|  | *Bacteroides.ovatus_391* | *Megasphaera.elsdenii_383* |
|  | *Eubacterium_402* | *Clostridiales_381* |
|  | *Bacteroides.massiliensis_12* | *Lactobacillus.iners_202* |
|  | *Lachnospiraceae_186* | *Clostridiales_272* |
|  |  | *Eubacterium.siraeum_104* |
|  |  | *Porphyromonadaceae_107* |
|  |  | *Firmicutes_106* |
|  |  | *Barnesiella.intestinihominis_101* |
|  |  | *Clostridia_295* |
|  |  | *Parasutterella.secunda_296* |
|  |  | *Clostridiales_299* |
|  |  | *Bacteroides.intestinalis_987* |
|  |  | *Barnesiella.intestinihominis_329* |
|  |  | *Pseudomonas.peli_328* |
|  |  | *Bacteria_325* |
|  |  | *Halomonas_320* |
|  |  | *Porphyromonadaceae_401* |
|  |  | *Bacillus.horikoshii_149* |
|  |  | *Eubacterium.coprostanoligenes_141* |
|  |  | *Bradyrhizobium.jicamae_140* |
|  |  | *Eubacterium.siraeum_48* |
|  |  | *Lachnospiraceae_45* |
|  |  | *Lachnoclostridium_248* |
|  |  | *Clostridiales_448* |
|  |  | *Megasphaera.elsdenii_447* |
|  |  | *Clostridiales_442* |
|  |  | *Acetanaerobacterium.elongatum_1003* |
|  |  | *Ochrobactrum.pseudogrignonense_216* |
|  |  | *Bacteroides_399* |
|  |  | *Alistipes.indistinctus_360* |
|  |  | *Sutterella.stercoricanis_151* |
|  |  | *Murdochiella.asaccharolytica_324* |
|  |  | *Haemophilus.parainfluenzae_19* |
|  |  | *Prevotella.disiens_49* |
|  |  | *Allobaculum.stercoricanis_508* |
|  |  | *Clostridium.ultunense_446* |
|  |  | *Barnesiella_215* |
|  |  | *Parasutterella.secunda_327* |
|  |  | *Coprococcus.catus_213* |
|  |  | *Coprobacillus.cateniformis_187* |
|  |  | *Paracoccus.sphaerophysae_506* |
|  |  | *Phascolarctobacterium.faecium_16* |
|  |  | *Aggregatibacter.segnis_218* |
|  |  | *Dialister.succinatiphilus_135* |
|  |  | *Clostridium.leptum_139* |
|  |  | *Klebsiella_102* |
|  |  | *Parabacteroides.distasonis_372* |
|  |  | *Prevotella.timonensis_99* |
| **Periphery** | | |
| **Shared Peripheries** | **Specific Peripheries** | |
|  | **Healthy** | **UC** |
| *Parabacteroides.gordonii_510* | *Bacteroides_985* | *Clostridiales_486* |
| *Dietzia_413* | *Ruminococcaceae_964* | *Mobiluncus.curtisii.holmesii_525* |
| *Atopobium.vaginae_411* | *Prevotella.copri_1042* | *Acinetobacter.venetianus_316* |
| *Ruminococcus.bromii_58* | *Kofleria.flava_524* | *Lactobacillus_317* |
| *Streptococcus.lutetiensis_59* | *Staphylococcus_312* | *Blastocatella.fastidiosa_419* |
| *Parabacteroides.merdae_54* | *Peptoniphilus.ivorii_313* | *Allobaculum.stercoricanis_555* |
| *Clostridiales_1034* | *Clostridiales.Family.XIII..Incertae.Sedis_315* | *Lachnospiraceae_777* |
| *Prevotella.stercorea_23* | *Leuconostoc.lactis_370* | *Lachnospiraceae_772* |
| *Tyzzerella.nexilis_22* | *Ruminiclostridium_371* | *Veillonella.dispar_51* |
| *Roseburia.inulinivorans_877* | *Lachnospiraceae_551* | *Bacteroides_53* |
| *Ruminococcus.lactaris_870* | *Blautia.faecis_679* | *Neisseria_250* |
| *Peptococcus.niger_583* | *Erysipelatoclostridium_55* | *Eubacterium.coprostanoligenes_255* |
| *Bacteroides_587* | *Dialister.succinatiphilus_251* | *Beijerinckiaceae_254* |
| *Blautia_1020* | *Alistipes.shahii_253* | *Anaerococcus.tetradius_257* |
| *Oscillibacter.valericigenes_129* | *Paraprevotella.clara_589* | *Bacteria_259* |
| *Faecalibacterium.prausnitzii_1074* | *Bacteroides.plebeius_989* | *Delftia_258* |
| *Bacteria_303* | *Prevotella_26* | *Peptoniphilaceae_28* |
| *Erysipelatoclostridium.ramosum_300* | *Dialister.propionicifaciens_177* | *Lactobacillus.rogosae_20* |
| *Clostridiales_264* | *Ruminococcus_172* | *Clostridium.lactatifermentans_176* |
| *Lactobacillus.rogosae_266* | *Clostridiales.Family.XIII..Incertae.Sedis_1083* | *Butyricicoccus.pullicaecorum_173* |
| *Peptoniphilus.gorbachii_112* | *Alistipes.onderdonkii_871* | *Parvimonas.micra_178* |
| *Clostridiales_118* | *Ruminococcus.faecis_780* | *Eubacterium.yurii.schtitka_518* |
| *Prevotella_281* | *Clostridiales_451* | *Eubacterium.dolichum_435* |
| *Parabacteroides.gordonii_31* | *Clostridiales.Family.XIII..Incertae.Sedis_454* | *Paraprevotella.clara_580* |
| *Tissierellia_160* | *Prevotella.copri_904* | *Lachnospiraceae_515* |
| *Bacteroides.ovatus_81* | *Parabacteroides.goldsteinii_222* | *Eubacterium.infirmum_453* |
| *Acidaminococcaceae_80* | *Caulobacter_226* | *Pyramidobacter.piscolens_452* |
| *Lachnoclostridium_977* | *Alistipes.indistinctus_350* | *Allobaculum.stercoricanis_456* |
| *Clostridium.symbiosum_332* | *Ruminococcus.flavefaciens_353* | *Prevotella.oralis_220* |
| *Clostridium.colinum_73* | *Clostridium.viride_642* | *Peptoniphilus.lacrimalis_223* |
| *Clostridiales_76* | *Porphyromonas_121* | *Eggerthella.lenta_428* |
| *Bacteroides.thetaiotaomicron_7* | *Clostridiales_63* | *Pedobacter.ruber_351* |
| *Prevotella.copri_5* | *Alistipes.putredinis_62* | *Tyzzerella_126* |
| *Bacteroides.dorei_3* | *Bacteria_460* | *Porphyromonadaceae_123* |
| *Porphyromonas_275* | *Lachnospiraceae_1027* | *Blautia.obeum_947* |
| *Finegoldia.magna_90* | *Eubacterium.xylanophilum_763* | *Blautia.luti_496* |
| *Prevotella_181* | *Prevotella.buccalis_115* | *Flavobacterium_495* |
| *Prevotella.stercorea_237* | *Bacteroides.caccae_110* | *Anaerococcus.octavius_462* |
| *Prevotella.copri_137* | *Ruminococcaceae_287* | *Bifidobacterium.animalis_461* |
| *Anaerococcus.vaginalis_93* | *Clostridiales_283* | *Proteobacteria_465* |
| *Phascolarctobacterium.faecium_131* | *Blautia.wexlerae_37* | *Psychrobacter.cryohalolentis_541* |
| *Paraprevotella.clara_88* | *Eubacterium.ventriosum_163* | *Pseudomonas_611* |
| *Eubacterium.desmolans_284* | *Firmicutes_309* | *Atopostipes.suicloacalis_307* |
| *Methylobacterium_201* | *Sutterella.wadsworthensis_165* | *Atopobium.minutum_306* |
| *Lactobacillus.rogosae_650* | *Clostridium.asparagiforme_999* | *Paraburkholderia.ferrariae_301* |
| *Bacteria_92* | *Ruminococcaceae_500* | *Peptostreptococcus.stomatis_114* |
| *Bacteroides.uniformis_39* | *Massilia.aerilata_504* | *Ruminococcaceae_282* |
| *Bacteroides.stercoris_14* | *Clostridiales_337* | *Bacteroides.cellulosilyticus_38* |
| *Clostridium.ventriculi_132* | *Brevundimonas_334* | *Cyanobacteria_34* |
| *Dorea.longicatena_100* | *Clostridiales_641* | *Ruminococcus.gnavus_32* |
| *Eubacterium.tortuosum_150* | *Clostridiales_349* | *Phenylobacterium_162* |
| *Ruminococcus.albus_74* | *Clostridiales_341* | *Flavonifractor.plautii_161* |
| *Alloprevotella.rava_185* | *Clostridiales.Family.XIII..Incertae.Sedis_344* | *Clostridium.aldenense_929* |
| *Bacteroides.dorei_182* | *Sutterella.stercoricanis_151* | *Clostridium.bolteae_86* |
|  | *Deltaproteobacteria_72* | *Bacteroides_89* |
|  | *Sutterellaceae_70* | *Ruminococcaceae_426* |
|  | *Eubacterium.ruminantium_79* | *Lachnospiraceae_424* |
|  | *Prevotella.copri_936* | *Gallicola.barnesae_422* |
|  | *Barnesiella.intestinihominis_231* | *Peptoniphilus_421* |
|  | *Barnesiella.intestinihominis_239* | *Prevotella.pallens_482* |
|  | *Clostridium.sardiniense_953* | *Vibrio.pelagius_338* |
|  | *Rhodospirillaceae_168* | *Peptoniphilus.methioninivorax_339* |
|  | *Lachnospiraceae_385* | *Bacteria_333* |
|  | *Clostridiales_476* | *Alloprevotella_330* |
|  | *Barnesiella.intestinihominis_273* | *Methylobacterium.suomiense_331* |
|  | *Clostridiales_272* | *Dokdonella.immobilis_439* |
|  | *Ruminococcus.flavefaciens_274* | *Bifidobacterium.longum_343* |
|  | *Eubacterium.siraeum_104* | *Howardella.ureilytica_347* |
|  | *Porphyromonadaceae_107* | *Enterobacteriaceae_345* |
|  | *Barnesiella.intestinihominis_101* | *Clostridiales_157* |
|  | *Ruminococcus.albus_291* | *Bilophila.wadsworthia_71* |
|  | *Sneathia.sanguinegens_196* | *Porphyromonas.asaccharolytica_77* |
|  | *Prevotella.copri_850* | *Clostridium.bolteae_75* |
|  | *Lachnospiraceae_534* | *Clostridiales_78* |
|  | *Succinivibrio.dextrinosolvens_409* | *Ruminiclostridium_236* |
|  | *Prevotella_406* | *Bacteroides.nordii_235* |
|  | *Alistipes.indistinctus_148* | *Kocuria.palustris_234* |
|  | *Eubacterium.coprostanoligenes_141* | *Clostridium.lactatifermentans_779* |
|  | *Bradyrhizobium.jicamae_140* | *Parabacteroides.distasonis_8* |
|  | *Alloprevotella.rava_43* | *Megamonas.rupellensis_6* |
|  | *Bacteroidetes_244* | *Streptococcus.infantis_1* |
|  | *Acetanaerobacterium.elongatum_1003* | *Chlamydia.muridarum_473* |
|  | *Prevotella.copri_697* | *Acetobacterium_474* |
|  | *Clostridium.leptum_145* | *Flavonifractor.plautii_577* |
|  | *Clostridium.methylpentosum_427* | *Clostridiales_752* |
|  | *Lachnospiraceae_395* | *environmental.samples_208* |
|  | *Butyricimonas_696* | *Campylobacter.ureolyticus_204* |
|  | *Clostridium.methylpentosum_700* | *Chryseobacterium.soli_373* |
|  | *Ruminiclostridium_369* | *Proteobacteria_376* |
|  | *Bacillus.horikoshii_149* | *Rhodococcus.yunnanensis_377* |
|  | *Clostridiales_199* | *Akkermansia.muciniphila_105* |
|  | *Holdemanella.biformis_366* | *Ruminococcaceae_108* |
|  | *Eubacterium.eligens_64* | *Negativicoccus.succinicivorans_290* |
|  | *Clostridiales_299* | *Macrococcus.caseolyticus_293* |
|  | *Firmicutes_749* | *Clostridiales_292* |
|  | *Ruminococcus_66* | *Murdochiella.asaccharolytica_298* |
|  | *Gemmiger.formicilis_69* | *Streptomyces.exfoliatus_494* |
|  | *Bacteroidales_120* | *Clostridium.cocleatum_492* |
|  | *Ochrobactrum.pseudogrignonense_216* | *Clostridiales_532* |
|  | *Alistipes.onderdonkii_85* | *Dialister.succinatiphilus_323* |
|  | *Coprococcus.catus_213* | *Acidimicrobiales_302* |
|  | *Moryella.indoligenes_227* | *Clostridia_404* |
|  | *Clostridiales_448* | *TM7_390* |
|  | *Microbacteriaceae_597* | *Megasphaera.micronuciformis_394* |
|  | *Alistipes.shahii_166* | *Alloprevotella.rava_47* |
|  | *Clostridiales_743* | *Gemella.morbillorum_242* |
|  | *Alistipes.indistinctus_386* | *Prevotella_247* |
|  | *Coprobacillus.cateniformis_187* | *Bacteroides.clarus_595* |
|  | *Fusobacterium.mortiferum_17* | *Dialister.micraerophilus_449* |
|  | *Rhodospirillaceae_246* | *Bacteria_440* |
|  | *Escherichia_2* | *Hymenobacter_441* |
|  | *Parabacteroides.gordonii_537* | *Fusobacterium_212* |
|  | *Eubacterium.coprostanoligenes_155* | *Rhodospirillum.centenum_209* |
|  | *Clostridium.perfringens_46* | *Lachnospiraceae_186* |
|  | *Clostridiales_364* | *Tyzzerella_193* |
|  | *Agrobacterium.rubi_362* | *Varibaculum.cambriense_616* |
|  |  | *Eubacterium_402* |
|  |  | *Bacteroides.massiliensis_12* |
|  |  | *Allobaculum.stercoricanis_374* |
|  |  | *Bacillus.vallismortis_415* |
|  |  | *Clostridium.puniceum_134* |
|  |  | *Burkholderia_294* |
|  |  | *Turicibacter.sanguinis_252* |
|  |  | *Bacteroides_862* |
|  |  | *Veillonella.atypica_470* |
|  |  | *Ruminococcaceae_224* |
|  |  | *Anaerostipes.butyraticus_164* |
|  |  | *Coprococcus.comes_363* |
|  |  | *Peptostreptococcaceae_35* |
|  |  | *Clostridium_271* |
|  |  | *Ruminococcus.callidus_189* |
|  |  | *Butyricimonas.virosa_192* |
|  |  | *Sphingomonadaceae_542* |
|  |  | *Lactobacillus.salivarius_198* |
|  |  | *Alloprevotella.tannerae_365* |
|  |  | *Prevotella.oris_445* |
|  |  | *Bacteroides.coprophilus_15* |
|  |  | *Eubacterium.ramulus_195* |
|  |  | *Methylomonas.scandinavica_536* |
|  |  | *Clostridiales_91* |
|  |  | *Anaerostipes.caccae_443* |
|  |  | *Prevotella.bivia_42* |
|  |  | *Lactobacillus.rogosae_886* |
|  |  | *Achromobacter_361* |
|  |  | *Lachnospiraceae_958* |
|  |  | *Peptoniphilus.asaccharolyticus_480* |
|  |  | *Aquabacterium.parvum_497* |
|  |  | *Clostridiales_276* |
|  |  | *Moraxella_240* |
|  |  | *Bacteroides.fragilis_10* |
|  |  | *Enterobacteriaceae_197* |
|  |  | *Dialister.pneumosintes_183* |

**Table S6. Basic network properties of the species co-occurrence networks (SCNs) of the mucosal microbiome**

| **Treatment** | **Num. of Nodes** | ***Num. of***  ***Edges*** | ***Average Degree*** | ***Avg. Local Cluster Coefficient*** | ***Diameter*** | ***Average Path Length*** | ***Connected Components*** | ***Network Density*** | ***Network Modularity*** | ***Num. of***  ***Communities*** |
| --- | --- | --- | --- | --- | --- | --- | --- | --- | --- | --- |
| Healthy | 316 | 995 | 6.297 | 0.442 | 14 | 5.324 | 15 | 0.020 | 0.709 | 35 |
| UC | 396 | 2115 | 10.682 | 0.466 | 17 | 4.717 | 8 | 0.027 | 0.596 | 46 |

**Table S7**. The strongly connected clusters in species co-occurrence networks (SCNs) of the mucosal microbiome

| **Healthy** | | | | **UC** | | | |
| --- | --- | --- | --- | --- | --- | --- | --- |
| ***Cluster No.*** | ***Score*** | ***Nodes*** | ***Edges*** | ***Cluster No.*** | ***Score*** | ***Nodes*** | ***Edges*** |
| 1 | 9.115 | 26 | 237 | 1 | 9.25 | 24 | 222 |
| 2 | 4.4 | 10 | 44 | 2 | 8.95 | 20 | 179 |
| 3 | 4 | 10 | 40 | 3 | 3.333 | 18 | 60 |
| 4 | 3.692 | 13 | 48 | 4 | 3.333 | 9 | 30 |
| 5 | 2 | 6 | 12 | 5 | 3 | 7 | 21 |
| 6 | 1.786 | 14 | 25 | 6 | 2.917 | 12 | 35 |
| 7 | 1.556 | 9 | 14 | 7 | 2.571 | 7 | 18 |
| 8 | 1.5 | 4 | 6 | 8 | 2.348 | 23 | 54 |
| 9 | 1.333 | 6 | 8 | 9 | 2 | 8 | 16 |
| 10 | 1.333 | 6 | 8 | 10 | 2 | 5 | 10 |
| 11 | 1.25 | 4 | 5 | 11 | 2 | 11 | 22 |
| 12 | 1 | 3 | 3 | 12 | 1.8 | 5 | 9 |
| 13 | 1 | 3 | 3 | 13 | 1.8 | 5 | 9 |
| 14 | 1 | 3 | 3 | 14 | 1.5 | 4 | 6 |
| 15 | 1 | 3 | 3 | 15 | 1.5 | 4 | 6 |
| 16 | 1 | 3 | 3 | 16 | 1.5 | 4 | 6 |
| 17 | 1 | 3 | 3 | 17 | 1.25 | 4 | 5 |
| 18 | 1 | 3 | 3 | 18 | 1.25 | 4 | 5 |
| 19 | 1 | 3 | 3 | 19 | 1 | 3 | 3 |
| 20 | 1 | 3 | 3 | 20 | 1 | 3 | 3 |
| 21 | 1 | 5 | 5 | 21 | 1 | 3 | 3 |
|  |  |  |  | 22 | 1 | 3 | 3 |
|  |  |  |  | 23 | 1 | 3 | 3 |

**Table S8***.* The shared and specific OTUs between the #1 cluster in the healthy mucosa SCN and the #2 cluster in the UC mucosa SCN

| **Shared OTUs** | **Specific OTUs** | |
| --- | --- | --- |
|  | **Healthy-SCN: #1 Cluster** | **UC-SCN: #2 Cluster** |
| *Acinetobacter_*1070 | *Bacteria_*259 | *Bradyrhizobium.jicamae_*140 |
| *Acinetobacter.baumannii_*229 | *Bacteria_*416 | *Brevundimonas_*334 |
| *Acinetobacter.junii_*83 | *Burkholderia_*294 | *Delftia_*258 |
| *Asticcacaulis.excentricus_*174 | *Burkholderiales_*572 | *Methylobacterium_*201 |
| *Bacillus.cereus_*18 | *Clostridiales_*78 | *Serratia.marcescens.sakuensis_*169 |
| *Beijerinckiaceae_*254 | *Cyanobacteria_*34 |  |
| *Carnobacterium.gallinarum_*138 | *Methylobacterium.jeotgali_*243 |  |
| *Exiguobacterium.aestuarii_*170 | *Moraxella_*240 |  |
| *Gammaproteobacteria_*24 | *Phenylobacterium_*162 |  |
| *Geobacillus.stearothermophilus_*36 | *Rhodococcus.jialingiae_*354 |  |
| *Lactococcus.lactis.hordniae_*153 | *Streptococcus.parauberis_*552 |  |
| *Lactococcus.piscium_*13 |  |  |
| *Pseudomonas_*210 |  |  |
| *Pseudomonas.lundensis_*128 |  |  |
| *Streptococcus.agalactiae_*113 |  |  |

**Table S9. Positive-to-negative links (P/N) ratios within whole network, main phyla, and core/periphery of each SCN**

| **Treatment** | | **Group** | **Positive Links (+)** | **Negative Links (-)** | **P/N (+/-) Ratio** |
| --- | --- | --- | --- | --- | --- |
| Whole network | | Healthy | 919 | 76 | 12.092 |
|  |  | UC | 2083 | 32 | 65.094 |
| **Phylum** | | | | | |
| *Bacteroidetes* | *Bacteroidetes* | Healthy | 102 | 1 | 102.000 |
|  |  | UC | 100 | 0 | Inf |
| *Bacteroidetes* | *Firmicutes* | Healthy | 160 | 7 | 22.857 |
|  |  | UC | 425 | 0 | Inf |
| *Bacteroidetes* | *Proteobacteria* | Healthy | 21 | 3 | 7.000 |
|  |  | UC | 68 | 1 | 68.000 |
| *Firmicutes* | *Firmicutes* | Healthy | 329 | 45 | 7.311 |
|  |  | UC | 822 | 12 | 68.500 |
| *Firmicutes* | *Proteobacteria* | Healthy | 129 | 17 | 7.588 |
|  |  | UC | 258 | 17 | 15.176 |
| *Proteobacteria* | *Proteobacteria* | Healthy | 80 | 0 | Inf |
|  |  | UC | 120 | 1 | 120.000 |
| **Core/Periphery** | | | | | |
| Core | Core | Healthy | 490 | 60 | 8.167 |
|  |  | UC | 1242 | 16 | 77.625 |
| Periphery | Periphery | Healthy | 148 | 7 | 21.143 |
|  |  | UC | 276 | 3 | 92.000 |
| Core | Periphery | Healthy | 281 | 9 | 31.222 |
|  |  | UC | 565 | 13 | 43.462 |

**Table S10*.* Information on four target OTUs, including *p*-values of significant differences in abundance and P/N (positive-to-negative links) ratios in healthy-SCN and UC-SCN**

| **Target OTU** | ***p*-value of significant difference in abundance** | | **Healthy-SCN** | | | **UC-SCN** | | |
| --- | --- | --- | --- | --- | --- | --- | --- | --- |
|  | **Healthy<UC** | **Healthy>UC** | **Positive Links (+)** | **Negative Links (-)** | **P/N (+/-) Ratio** | **Positive Links (+)** | **Negative Links (-)** | **P/N (+/-) Ratio** |
| *Clostridium tertium*_175 | 0.031 | 0.969 | 2 | 12 | 0.167 | 3 | 1 | 3.000 |
| *Odoribacter splanchnicus*_221 | 1.000 | 0.000 | 5 | 11 | 0.455 | 10 | 0 | Inf |
| *Flavonifractor plautii*_161 | 0.049 | 0.951 | 2 | 9 | 0.222 | 4 | 0 | Inf |
| *Ruminococcus gnavus*_32 | 0.032 | 0.968 | 0 | 6 | 0.000 | 3 | 2 | 1.500 |
